# Information needs of physicians regarding the diagnosis of rare diseases: a questionnaire-based study in Belgium

**DOI:** 10.1186/s13023-019-1075-8

**Published:** 2019-05-04

**Authors:** Liese Vandeborne, Eline van Overbeeke, Marc Dooms, Birgit De Beleyr, Isabelle Huys

**Affiliations:** 1 Anticancer Fund, Boechoutlaan 221, Strombeek-Bever, Belgium; 2 Clinical Pharmacology and Pharmacotherapy, KU Leuven, Herestraat 49, Box 521, Leuven, Belgium; 3 Janssen-Cilag NV, Antwerpseweg 15-17, Beerse, Belgium

**Keywords:** Rare diseases, Information needs, Physicians, Orphanet, Diagnosis, Red flags

## Abstract

**Background:**

Late and misdiagnoses of rare disease patients are common and often result in medical, physical and mental burden for the patient, and financial and emotional burden for the patient’s family. Low rare disease awareness among physicians is believed to be one of the reasons for these late and misdiagnoses of rare disease patients. The aim of this study was to investigate how information and education could be tailored to the needs and preferences of physicians in Belgium to increase their rare disease awareness and support them in diagnosing patients with a rare disorder. Nine exploratory interviews with Belgian rare disease experts were performed in December 2016 to help the development of a questionnaire on information needs of physicians and their consulted information sources in rare disease awareness and diagnosis. This online questionnaire was then completed by Belgian physicians (*n* = 295), including general practitioners (GPs), pediatricians and other specialists (i.e. neurologists, pediatric neurologists, endocrinologists and pediatric endocrinologists) during January and February 2017.

**Results:**

Rare disease knowledge and awareness were the lowest among GPs and the highest among specialists. Interviewed experts indicated that physicians’ academic and continuous medical education should be focused more on “red flags” to increase rare disease attentiveness in daily clinical practice. GPs scored their academic education on rare diseases as insufficient but pediatricians and other specialists scored it significantly better (*p* < 0.001). Even though GPs declared to only need information on rare diseases when having a rare disease patient in their practice, specialists indicated to need more rare disease information in general. Most physicians confirmed that they had specific information needs regarding rare diseases. Unlike specialists, the majority of GPs were unaware of information sources such as Orphanet.

**Conclusion:**

In order to effectively support physicians in Belgium to diagnose rare diseases early, the academic medical education on rare diseases should be revised. Teaching methods should be focused more on casuistry and “red flags”. An Orphanet-like digital platform about rare disease symptoms, diagnostic tests and reference centers might be ideal to support correct and timely diagnosis.

**Electronic supplementary material:**

The online version of this article (10.1186/s13023-019-1075-8) contains supplementary material, which is available to authorized users.

## Background

The diagnosis of a rare disease begins like every other diagnosis: a patient with clinical symptoms will consult his/her general practitioner (GP) or, if the patient is a child, its parents might opt to consult a pediatrician as primary health care professional (PHCP) [1, 2]. PHCPs will diagnose and treat the patient themselves or will refer him/her to a HCP specialized in that particular pathology [3, 4]. Worsening symptoms, occurrence of new symptoms, ineffectiveness of prescribed treatments or unexplainable progression of the disease are arguments for referring the patient back and forth between PHCPs and specialists in what is called, a referral loop or “*Artze-Odyssey*” [5, 6].

Diagnostic referral loops are not only a source of frustration for HCPs, but also allow disease progression and symptom worsening, which can conflict with possible treatment options [5, 7]. Besides medical, physical and mental burden for the patient, families can suffer economic losses and experience financial problems because of visits to many different physicians, intermediate care and diagnostic testing. Frustration, stress, anxiety, self-doubt and emotional trauma are consequences of diagnostic incompetence and ineffective patient care, seriously affecting families’ relationships with physicians and other HCPs [8].

The time between the occurrence of first symptoms and the diagnosis is important in every disease but most often problematic in rare diseases [9]. This time delay feeds the diagnostic referral loop, which is typical for rare diseases and known to cause late and misdiagnosis. A survey among 631 rare disease patients living in the United Kingdom (UK) and the United States of America (USA) indicated that the average time to diagnose a rare disease patient correctly varied between 5.6 and 7.6 years [10]. Genetic Alliance UK reported that 1 in 3 rare disease patients has to wait over 2 years to receive the correct diagnosis [11]. The King Baudouin Foundation reported that in Belgium, 22% of rare disease patients visited more than 5 physicians before they received a confirmation of the proper diagnosis, 7% visited more than 10 physicians, 44% was misdiagnosed at first and 75% of these misdiagnosed patients received inappropriate and ineffective treatment [12].

Low rare disease awareness and knowledge among HCPs is believed to be one of the reasons why many rare disease patients face late and misdiagnoses [5, 13]. Especially PHCPs like GPs, pediatricians and pharmacists, who are usually the first to identify health problems in patients, appear to have difficulties with raising the clinical suspicion for a rare disease diagnosis. PHCPs could fundamentally contribute to facilitating the rare disease patient journey by thinking about rare diseases when encountering an “unusual” patient [14–16]. Both education and information about rare diseases might contribute to raising awareness on and clinical suspicion of rare disease diagnoses. In the past, it has been shown that the education of medical students on rare disease symptoms is insufficient and does not allow diagnosis of rare diseases [17].

Studies on physicians’ preferences regarding education on rare diseases have been performed in countries like Sweden and Spain, but not in Belgium [18, 19]. This study investigates how information and education could be tailored to the needs and preferences of physicians in Belgium to increase their rare disease awareness and support them in diagnosing rare disease patients. These concepts, education and information, were not taken up in the Belgian national plan for rare diseases (https://www.eurordis.org/). Education and information needs may be considered as generally known but seldom investigated and published. Since neurological and endocrine rare disorders are two large rare disease groups, according to the Orphanet database [20], and three quarters of rare disease patients are children [21], this study focused on neurological, endocrine and pediatric rare disorders.

## Methods

Exploratory interviews with rare disease experts were conducted. Based on the findings from the interviews, a questionnaire was setup to investigate the level of awareness and knowledge on rare diseases, and information needs among Belgian GPs, pediatricians, neurologists, endocrinologists, pediatric neurologists and pediatric endocrinologists.

### Exploratory interviews

The interviews were conducted in December 2016 with 9 Belgian rare disease experts, identified through purposive sampling and snowballing. The purpose of the interviews was to inform the set-up of the survey and not to obtain data saturation. A Dutch and French interview guide was set up for in-depth interviews, consisting of four open questions and several sub questions (Additional file 1). The role of rare disease awareness in late and misdiagnosis, the contribution of academic and continuous medical training to rare disease awareness, and the value of specific rare disease information sources to facilitate the diagnosis of rare diseases were questioned. Informed consent was obtained from all interviewees. The interviews, taking 45 to 60 min, were audio-recorded and transcribed *verbatim*. Transcripts were not reviewed by the participants and were pseudonymized. The interviews were analyzed according to the Grounded Theory Approach and the qualitative analysis guide of KU Leuven (QUAGOL) with the QSR NVivo 10 software as Britten et al. described [22, 23].

### Questionnaire

A questionnaire was designed in French and Dutch (Additional file 2). Belgian GPs, pediatricians, neurologists, endocrinologists, pediatric neurologists and pediatric endocrinologists were invited to participate in the study. Participants had to belong to one of the target groups, be of age and competent to give informed consent. Target group members were invited via e-mail or through a specific physicians’ association, via e-mail, websites or newsletters. These associations included around ninety GP groups, the Flemish association for pediatrics (‘Vlaamse Vereniging voor Kindergeneeskunde’) with 243 members, the Belgian society of French speaking pediatricians (‘Groupement Belge des Pédiatres de langue Française’) with less than 500 members, the Belgian Society of Neurology with around 300 members, the Belgian Society for Pediatric Endocrinology and Diabetology with 38 members, the WIV-ISP who contacted 198 physicians, and the endocrinology and neurology departments of Belgian hospitals.

The questionnaire consisted of 25 multiple choice questions divided into five parts. The first part investigated characteristics of participants and the second knowledge on rare diseases. Between part 2 and 3, the EU definition of rare diseases was given to ensure every participant had the same level of knowledge. Part 3 investigated rare disease awareness. Between part 3 and 4, a broader explanation of rare diseases was provided and some characteristics were highlighted. Part 4 investigated academic and continuous education on rare diseases. The last part investigated information needs of participants regarding rare and ultra-rare diseases.

The questionnaire could only be completed after giving informed consent and participation was anonymous. Participants could access and complete the questionnaire online during January and February 2017. Statistical testing was performed using PC SAS version 9.4. Categorical data were tested with the chi-square test to reveal statistical differences (*p* < 0.05). If sample sizes were too small to perform chi-square tests, exact tests/*p*-values were used: the Fisher’s exact test for multinomial variables, and the Mantel-Haenszel chi-square test for ordinal variables.

## Results

### Exploratory interviews

A total of five medical specialists active in the field of rare diseases, two GPs with a specific interest in rare diseases, one nurse daily encountering rare disease patients in a specialized center and one advisor of health policy in Belgium were interviewed as rare disease experts. All experts recognized that efforts in the field of rare diseases are necessary to facilitate fast and correct diagnosis. In response to interview questions, experts mentioned numerous issues that should be considered when developing and implementing a rare disease information policy: issues relating to the structure of the healthcare system such as physician-physician interaction, physician-patient interaction and multidisciplinary diagnosis, and issues relating to information sources.

#### The patient as source of information

A thorough and well-interpreted anamnesis in first and second line health care is crucial to request adequate diagnostic tests, refer the patient correctly and refine the differential diagnosis accurately according to the experts. Every aspect, feeling and symptom of a patient and his medical file are important for physicians to be able to detect “red flags” and raise the suspicion of a rare disease. Detecting a “red flag” means that a physician feels something is not right, something more is going on with a patient than meets the eye or the combination of symptoms and test results are strange or abnormal, which leads to the consideration of a rare disease diagnosis. Even though some rare diseases’ pathophysiology is not well known today, making diagnosis difficult and leading to diagnostic delays and misdiagnosis, this type of diagnostic delay must be distinguished from the delay that could have been avoided if the treating physician was better informed about and more attentive towards rare diseases and “red flags”. In order to facilitate rare disease diagnosis, one expert suggested that diagnostic lab tests should be equipped with a system that analyzes the test results and suggests a differential diagnosis based upon “red flags”. These test results and proposed rare disease differential diagnosis could then be checked very carefully by the concerned physician taking the patient’s anamnesis into account, which might lead to the confirmation of a rare disease diagnosis.

#### Academic and continuous education

All experts agreed that physicians must be educated on rare diseases during medical school and continuous medical training. According to the experts, not only specialists but also GPs, who are focused on diagnosing and treating common pathologies, should be instructed about rare diseases. Extensive knowledge on rare diseases cannot be expected from medical students, but they should at least be told about rare diseases in general and in sub-discipline courses to make them more aware. When talking about continuous training sessions, most of the experts believed that sessions about a specific type of pathology that are mainly focused on common diseases should use the opportunity to mention some rare diseases that fit the scope of the session too. According to the experts, a multidisciplinary teaching method that focuses on casuistry, enables professors or other teachers to walk the audience (i.e. medical students or practicing physicians) through a realistic diagnostic journey: from patient anamnesis to establishing a differential diagnosis further to interpreting diagnostic test results and defining the correct diagnosis or care for a specific (type of) patient(s). Experts expressed that a teaching method starting at the level of the patient and leading towards a diagnosis is necessary, as most training sessions today start with the description of a disease followed by an enumeration of possible patient characteristics and symptoms. Based on patient characteristics and prior knowledge, professors must trigger medical students to reason outside the normal thinking and draw their attention to “red flags”, according to one of the experts. “Red flag” attentiveness can redirect a diagnosis positively and facilitates defining the correct (rare disease) diagnosis in the end. According to some of the experts, the need to be attentive to “red flags” should also be highlighted in continuous training sessions to trigger physicians in the audience to use another approach towards some of their own “unusual” patients.

#### Internet resources

The majority of experts believed that only specialized or university specialists, but not general specialists and GPs are aware of Orphanet, an internationally acknowledged online portal for reference information about rare diseases and orphan drugs. Some experts indicated that due to the way that Orphanet is organized today, it only targets physicians that are engaged in rare diseases and not those in search of a rare disease diagnosis. As additional information sources are unnecessary, existing information sources such as Orphanet could be further expanded, combined into one digital platform or combined into one comprehensive rare disease search engine, according to the experts. Such a search engine or digital platform must be used by all physicians and therefore taught and utilized in medical school as a tool supporting physicians in diagnosing patients correctly and referring them appropriately. According to most experts, physicians should be able to put patient symptoms and test results into this tool and get a differential diagnosis of rare diseases as output. Additionally, to shorten the list of differential diagnoses and refine the diagnostic search, one expert stated that the online tool must also make suggestions about extra diagnostic tests that could be performed or specific questions the physician could ask the patient. On top of a rare disease differential diagnosis, possible treatment options, contact details of acknowledged experts and reference centers or patients’ associations could be valuable output as well. The ideal tool should be up to date, freely available in the physicians’ language of choice and validated by numerous rare disease specialists and experts. Only then the tool will be capable of redirecting and refining diagnosis towards a rare disease, shorten time till diagnosis and prevent misdiagnosis. Most of the experts indicated that if medical students learned to work with such a tool, the tool could become very useful and supportive during their later career when confronted with rare diseases.

### Questionnaire

In total, 295 physicians completed the questionnaire. Most physicians that participated were GPs (39%), 32% were pediatricians, 9% were other specialists treating children (referred to as “pediatric specialists”) and 16% were specialists treating adult patients (referred to as “adult specialists”) (Table 1). Amongst adult specialists, 31 neurologists and 17 endocrinologists participated. Apart from general pediatricians, 12 pediatric neurologists and 15 pediatric endocrinologists participated. Eleven other specialists that completed the questionnaire, did not belong to one of the physician target groups and therefore their answers were not considered during further questionnaire analysis.

**Table 1:** Participant characteristics

Characteristic	Participants (n = 295)	
	*n*	*%*
Sex		
Female	183	62%
Male	112	38%
Age		
< 30 yr.	46	16%
30 to 45 yr.	101	34%
45 to 60 yr.	98	33%
> 60 yr.	50	17%
Profession		
General Practitioner	114	39%
Pediatrician	95	32%
Adult specialist	48	16%
Endocrinologist	17	6%
Neurologist	31	11%
Pediatric specialist	27	9%
Pediatric endocrinologist	15	5%
Pediatric neurologist	12	4%
Other	11	4%
Graduated at university in …		
Flanders	147	52%
Brussels	47	16%
Wallonia	93	32%
Working region		
Flanders	149	52%
Brussels	60	21%
Wallonia	78	27%
Workplace		
University hospital	90	31%
Peripheral hospital	65	22%
Outside hospital	129	44%
Other	10	3%

#### Knowledge

**Fig. 1:**
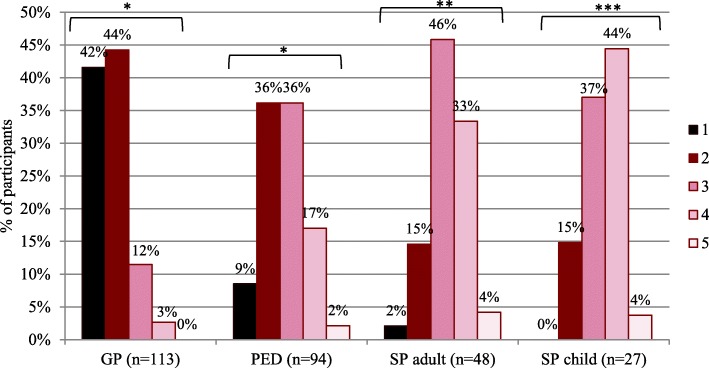
Physicians’ self-evaluated level of rare disease knowledge. Response options for the question ‘How do you assess your own knowledge about rare diseases?’ ranged from 1 (‘Poor’) to 5 (‘Excellent’); 2 = ‘Substandard’, 3 = ‘Average’ and 4 = ‘Good’. Statistical analysis was performed with Mantel-Haenszel Chi^2^ Test: *, significant different distribution of responses (*p* < 0.05) from this physician group compared to those from all other physician groups; **, significant different distribution of responses (p < 0.05) from SP adult compared to GP and PED while no difference with SP child was detected; ***, significant different distribution of responses (p < 0.05) from SP child compared to GP and PED while no difference with SP adult was detected. PED = pediatrician. SP adult = specialists treating adults (endocrinologists and neurologists). SP child = specialists treating children (pediatric endocrinologists and neurologists)

In the first part of the questionnaire, participants were asked to score their knowledge about rare diseases from 1, being poor, to 5, being excellent. Most GPs chose response options 1 and 2 which was statistically different from the other participants (*p* < 0.001), meaning their self-reported knowledge on rare diseases is poorer than that of the other physician groups (Fig. 1). Pediatricians were more likely to select option 2 and 3 compared to the other physician groups (*p* < 0.001), indicating their self-reported knowledge is better than that of GPs but worse than that of other specialists, as adult and pediatric specialists mostly chose option 3 and 4. Specialists rated their knowledge better compared to GPs and pediatricians (p < 0.001) but there was no difference detected in pediatric and adult specialists’ responses to this question. The answers of GPs to the questions regarding the EU definition of a rare disease, the characteristics of rare diseases and the correct orphan drug description, were significantly less correct than those of pediatricians, (pediatric) neurologists and (pediatric) endocrinologists (*p* < 0.05) (Additional graphs: figure III.II, III.III, III.IV, III.V).

#### Awareness

**Fig. 2:**
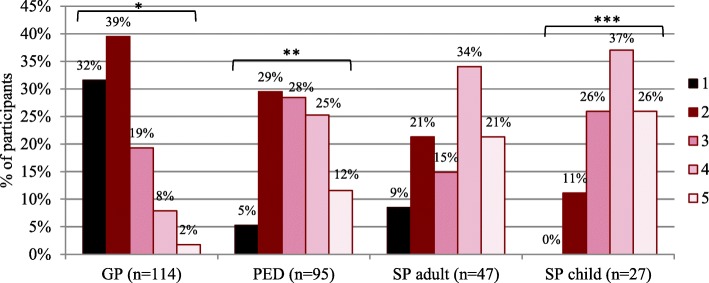
Physicians’ self-evaluated level of rare disease awareness. Response options for the question ‘Are you aware of rare diseases?’ ranged from 1 (‘Absolutely not’) to 5 (‘Definitely, yes’); 2 = ‘Poorly aware’, 3 = ‘Moderately aware’ and 4 = ‘Well aware’. Statistical analysis was performed with Mantel-Haenszel Chi^2^ Test: *, significant different distribution of responses (*p* < 0.001) from this physician group compared to those from all other physician groups; **, significant different distribution of responses (p < 0.05) from PED compared to GP and SP child while no difference with SP adult was detected; ***, significant different distribution of responses (p < 0.05) from SP child compared to GP and PED while no difference with SP adult was detected. PED = pediatrician. SP adult = specialists treating adults (endocrinologists and neurologists). SP child = specialists treating children (pediatric endocrinologists and neurologists)

The majority of GPs indicated to be absolutely not or only poorly aware of rare diseases (32 and 39% resp.) (Fig. 2). Pediatricians’ responses varied. A quarter of pediatricians indicated to be well aware (25%), a quarter indicated to be moderately aware (28%) and another quarter indicated to be poorly aware (29%). Of adult and pediatric specialists, 55 and 63% indicated to be well aware or definitely aware of rare diseases. The distribution of GPs’ answers was statistically different from the other participants (*p* < 0.001). Most participants estimated that there are less than 100,000 patients in Belgium that suffer from a rare disease (Additional graphs: figure III.VII), which does not correspond to the estimated number of 660,000 to 880,000 rare disease patients in Belgium according to literature [21]. On the question ‘Have you ever suspected that a patient suffered from a rare disease?’, almost all adult specialists (94%), pediatric specialists (100%) and pediatricians (72%) answered ‘Yes, multiple times.’ (figure III.X, appendix III). The responses of GPs varied but 52% had at least once suspected a patient to be suffering from a rare disease.

#### Academic and continuous medical education

**Fig. 3:**
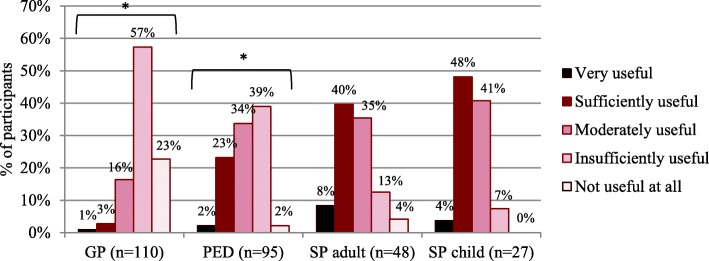
Physicians’ evaluation of the usefulness of their academic training for diagnosing rare diseases in daily practice. Response options for the question ‘How useful was your academic medical education for diagnosing rare diseases in daily practice?’. Full response options: *very useful*, I’m sure of myself when encountering rare diseases; *sufficiently*, I have got sufficient knowledge and training to diagnose rare disease patients; *moderate*, my academic medical training did not prepare me perfectly regarding rare disease diagnosis; *insufficiently*, I’d rather refer the patient to another specialist-doctor; *not useful at all*, the academic medical training did not prepare me concerning rare diseases. Statistical analysis was performed with Mantel-Haenszel Chi^2^ Test: *, significant different distribution of responses (*p* ≤ 0.001) from this physician group compared to those from all other physician groups. PED = pediatrician. SP adult = specialists treating adults (endocrinologists and neurologists). SP child = specialists treating children (pediatric endocrinologists and neurologists)

When participants were asked if their academic medical training dedicated a lot of time to rare diseases, 52% answered ‘No’ (Additional graphs: figure III.XII A). The majority of GPs (64%) and pediatric specialists (52%) opined there was not a lot of time spent on rare disease in their academic training (Additional graphs: figure III.XII B). GPs were more likely to answer ‘No’ than pediatricians (*p* = 0.004) and adult specialists (*p* = 0.002). The majority of pediatricians (56%) and adult specialists (63%) believed that a lot of time was spent on rare diseases during their academic training (Additional graphs: figure III.XII B). Twenty-nine percent of GPs, 44% of pediatricians, 39% of adult specialists and 44% of pediatric specialists indicated that more time must be spent on rare diseases during the academic medical training (Additional graphs: figure III.XII C). Only 8% of GPs, 3% of pediatricians and 4% of pediatric specialists believed too much time was dedicated to rare diseases during their academic medical training (Additional graphs: figure III.XII D). When asked how useful the academic medical training of participants was for diagnosing rare diseases in daily practice, the participants answered variously (Fig. 3). Compared to pediatricians and specialists, the distribution of GPs’ answers was statistically different (*p* < 0.001). Of GPs, 57% indicate that their medical training was insufficient and 23% that it was not at all useful in diagnosing rare diseases. Of pediatricians, 39 and 34% rated the academic training as insufficiently or moderately useful. The distribution of pediatricians’ answers was statistically different compared to all other participants (*p* ≤ 0.001). Adult and pediatric specialists answered the question ‘How useful was your academic medical training for diagnosing rare diseases in daily practice?’ similarly (*p* = 0.668), but were compared to GPs and pediatricians more likely to select the answer ‘Sufficiently’ (40 and 48% respectively) (p ≤ 0.001). Physicians graduated at a Walloon university were more likely to indicate their academic medical training regarding rare disease diagnostics as less sufficient than physicians graduated in Flanders (*p* = 0.006) or Brussels (*p* = 0.020) (Additional graphs: figure III.XIV). Difference was found in rating of academic training between physicians graduated in Flanders or Brussels (*p* = 0.968). Participants were asked if they would be interested in continuous training sessions about rare diseases. Almost two in five GPs indicated to be interested in sessions that combine common and rare disease topics (39%) (Additional graphs: figure III.XV). A minority of GPs (20%) was only interested in common disease topics. The distribution of GPs’ answers was statistically different from all other participants (*p* < 0.001). Pediatricians and adult and pediatric specialists (3, 0 and 0% resp.) seemed less interested in sessions about common disease topics compared to GPs (20%) and were more likely to have already attended rare disease sessions (32, 97 and 56% resp.) compared to GPs (3%).

#### Information needs

Most GPs (73%) did not know any specific rare disease information sources (Table 2). One in five GPs (22%) was familiar with Orphanet, as opposed to a clear majority of pediatricians (85%), adult specialists (75%) and pediatric specialists (89%). Apart from Orphanet, pediatricians and specialists also knew the Orphanet Journal of Rare Diseases, Belgian websites on rare diseases www.weesziekten.be/www.maladiesrares.be and EURORDIS. When participants were asked if they needed rare disease information, 83% of the GPs said ‘Yes’ (Additional graphs: figure III.XVI). Also 95% of pediatricians (PED), 97% of adult specialists (SP adult) and 100% of pediatric specialists (SP child) indicated to have information needs on rare diseases. The distribution of GPs’ answers was statistically different from the other participants (*p* < 0.001). In addition, 39% of GPs, 19% of pediatricians, 13% of adult and 15% of pediatric specialists indicated to only need information when they treat a rare disease patient themselves. One in five GPs (22%) and 15% of pediatricians and adult specialists admitted not to know where to find correct rare disease information. Sixteen percent of GPs answered ‘No’ to the question ‘Do you need information about rare diseases?’ mostly arguing that they do not have time left to dedicate to rare diseases. Less than 5% of pediatricians and specialists answered that they do not need rare disease information. When participants were asked if they had information needs regarding ultra-rare diseases, 77% of the GPs said ‘Yes’ (Additional graphs: figure III.XVII). Amongst pediatricians, 90% indicated to have information needs about ultra-rare diseases, amongst adult specialists 96% did and 93% of pediatric specialists. Amongst pediatricians and adult and pediatric specialists, 19, 27 and 26% indicated to be interested in information about ultra-rare diseases but only when they treat such a patient, compared to 46% of GPs. Around 40% of pediatricians and specialists scored their information needs regarding rare diseases equal to the needs regarding ultra-rare diseases, compared to 18% of GPs. More GPs (23%) indicated not to need information about ultra-rare diseases compared to pediatricians and specialists (10, 4 and 7% resp.). The distribution of GPs’ answers was statistically different from the other participants (*p* < 0.05). Next, participants were asked what kind of rare disease information they needed or wanted to receive. The information subjects GPs chose most were prevention and screening of rare diseases (46%), patient referral (63%), differential diagnosis (64%) and rare disease symptoms (71%) (Additional graphs: figure III.XVIII). These four subjects were also the ones most selected by pediatricians (67, 65, 86 and 90% resp.). Of pediatricians, 16% indicated that they only wanted to receive information about rare pediatric diseases. Most of specialists treating adults and children also indicated to need information on prevention and screening (58 and 63%), disease symptoms (75 and 74%) and differential diagnosis (83 and 74%). Furthermore, specialists indicated to need information about orphan drugs (50 and 41%) and rare disease treatment (42 and 33%). Two in five specialists indicated only to need information about rare diseases that relates to the specialism they are working in, i.e. endocrinology or neurology (44 and 41% resp.). Whether or not rare diseases are curable today, does not influence the information needs of participants, as only 1% of GPs, 6% of pediatricians, 4% of adult specialists and 0% of pediatric specialists selected to need information only about rare diseases that are curable. Altogether, participants seemed less interested in information about fundamental and translational research, physicians diagnosing rare diseases and their clinical cases, how to deal with rare disease patients and patient journeys (Additional graphs: figure III.XVIII). Finally, participants were asked how they wanted to receive rare disease information. Information channels selected by the majority of all participants were continuous training sessions (GPs 60%, PED 76%, SP adult 79% and SP child 59%) and experts or centers of expertise (GPs 51%, PED 57%, SP adult 52% and SP child 63%) (Additional graphs: figure III.XIX). The response option ‘Congresses, symposia and guest lectures’ was selected by the majority of pediatricians (80%) and specialists (77 and 85%) but not by the majority of GPs (34%). The majority of pediatricians wanted to receive rare disease information through physicians’ associations (53%) or during the academic specialist training (55%). Less popular information channels were online videos, brochures and information leaflets, journals, magazines and publications, information kits, mobile applications, e-mails, websites or digital platforms, advertising campaigns, basic medical academic training, orphan drug manufacturers, patients, patients’ associations and sickness funds.

**Table 2:** Results for the question: ‘Which of the listed organizations, search engines and information sources about rare disease do you know?’

Source of information	GP	PED	SP adult	SP child
	*n = 110*	*n = 93*	*n = 48*	*n = 27*
RaDiOrg	1%	5%	8%	19%
RDB	1%	5%	8%	15%
EURORDIS	0%	9%	10%	26%
Orphanet	22%	85%	75%	89%
Global Genes	1%	8%	4%	11%
FindZebra	1%	1%	8%	7%
www.weesziekten.be/ www.maladiesrares.be	5%	8%	15%	11%
NORD	0%	9%	8%	4%
ICORD	0%	2%	6%	7%
Orphanet Journal of Rare Diseases	2%	37%	33%	78%
None of the above	73%	14%	19%	7%

*PED* Pediatrician, *SP adult* Specialists treating adults (endocrinologists and neurologists), *SP child* Specialists treating children (pediatric endocrinologists and neurologists)

## Discussion

### Knowledge

Experts interviewed in this study believe that the basic knowledge of physicians in Belgium about rare diseases is low, particularly among GPs. Questionnaire results confirm this statement, as 86% of GPs indicated their knowledge is substandard or poor. Of pediatricians, 72% rated their knowledge as substandard or average and around 80% of adult and pediatric specialists indicate their knowledge is average or good. Questionnaire results indicate as well that, when compared to the worldwide population of physicians that participated in the study of Engel et al. [7], more GPs in Belgium believe their knowledge on rare diseases is poor (86% vs. 56%) and less specialists in Belgium believe their knowledge is good (80% vs. 94%).

### Awareness

Similar to the level of rare disease knowledge, more GPs than pediatricians rated their level of rare disease awareness as poor and more specialists than pediatricians rated their level of awareness as good. Even though a lot of GPs indicated not to be well aware of rare diseases and know typical rare disease characteristics, half of the GPs already suspected at least once that a patient was suffering from a rare disease, which strengthens the conclusion of Buntinx et al. [15] that GPs play an important role in rare disease diagnosis and thus should be better supported in detecting rare diseases. Most physicians are not able to estimate correctly how many patients in Belgium are affected by rare diseases. The underestimation of the number of rare disease patients might indicate a low level of rare disease awareness.

### Information needs

As in Gavhed et al. [13] who reported that Swedish HCPs do not know where to find correct rare disease information, questionnaire results revealed that 22% of GPs and 15% of pediatricians and specialists in Belgium do not know where to find correct rare disease information. Moreover, most physicians that completed the questionnaire indicated to need information about rare diseases. Half of GPs indicated to only require rare disease information when they have such a patient in their practice. GPs and pediatricians indicated to need rare disease information about prevention and screening, patient referral, differential diagnosis and rare disease symptoms. Specialists need information about prevention and screening, disease symptoms and differential diagnosis too, but indicated to need information about orphan drugs and rare disease treatment as well. Compared to GPs and pediatricians, specialists need less information about how to refer patients with a suspected rare disease and need more information about how to treat such a patient, which might be explained by the fact that specialists are diagnosing and treating most rare disease patients. Nevertheless, interviewed experts indicate that specialists should be informed about referring patients as well, since the choice to refer a patient to a center of expertise should be well thought through to enable these centers to be focused on true rare disease cases and provide the best possible care for the patient. Preferences for information channels to distribute the needed rare disease information are quite similar among the several types of physicians; the overall majority prefers expert centers and continuous training sessions. The majority of pediatricians also prefer to receive rare disease information during academic specialist training sessions and via physicians’ associations. In addition, pediatricians and other specialists are both interested in congresses, symposia and guest lectures for rare disease information dissemination as well.

### Information source

According to interviewed experts, the most typical rare disease information source is Orphanet. Interviewed experts predicted questionnaire results which show that only the majority of pediatricians and specialists know Orphanet and not GPs. Orphanet is not an ideal information source, according to these experts, but is a good basis to start from and perhaps to merge with other existing online information sources. Experts claim that physicians need a tool in which they can put patient symptoms and test results and retrieve a rare disease differential diagnosis as output. On top of a rare disease differential diagnosis, possible treatment options, contact details of acknowledged experts and reference centers or patients’ associations could be valuable output as well, according to interviewed experts. The ideal information source should be an up-to-date digital platform, freely available in physicians’ language of choice and validated by numerous rare disease specialists and experts. Even though physicians did not indicate websites or digital platforms as their rare disease information channel of choice, experts are convinced that such a digital platform could be very useful for all physicians.

### Education

Interviewed experts agree that the current rare disease academic education of physicians is not sufficient. Interviewed experts indicate that both specialists and GPs in training should be instructed about rare diseases. Questionnaire results indicate that less GPs (4%), pediatricians (25%), adult specialists (48%) and pediatric specialists (52%) in Belgium rate their medical training as (very) sufficiently useful, than reported in Engel et al. [5] in 2013 where 43% of GPs and 60% of specialists interviewed worldwide rated their medical training as efficient. More physicians graduated in Wallonia did not indicate that their education was useful regarding rare disease diagnosis in daily practice, compared to physicians graduated in Brussels or Flanders. This difference might indicate that medical rare disease education is poorer in Walloon universities than in universities in the rest of Belgium. Most experts believe that rare diseases should be addressed during academic medical education and during continuous training; not in courses or sessions fully dedicated to rare diseases but rather integrated in sub-discipline courses. Physicians that completed the questionnaire also indicate not to be interested in continuous training courses fully dedicated to rare diseases. Two in five GPs show interest in courses that combine common and rare disease topics even though one in five is only interested in sessions about common diseases. Most pediatricians and specialists have already attended rare disease continuous training sessions, explaining why they reported better knowledge and awareness on rare diseases compared to GPs. Training sessions in academic and continuous training should, according to interviewed experts, use casuistry as teaching method, ensuring such sessions start at the level of the patient and lead towards a rare disease diagnosis. In the meanwhile, session attendees should be educated on how to recognize “red flags”. Medical students should also be taught about these “red flags”, according to the experts.

### Limitations and future perspectives

There are several limitations to this study, which are important when interpreting the results. Not all medical specialties were targeted in this study, as only GPs, pediatricians, pediatric endocrinologists, endocrinologists, pediatric neurologists and neurologists could participate in the questionnaire. Targeting specific medical specialties allowed to formulate a specialty-specific question in the questionnaire and to increase the response rate by using a more direct and personal approach. Also, the distribution of questionnaire participants’ sex and age did not correspond with the sex and age distribution of the real physician population as described in the statistical report of the federal government about HCPs registered in Belgium in 2015 [24]. As 62% of the questionnaire participants were women and the real population of GPs, pediatricians, (pediatric) endocrinologists and (pediatric) neurologists exists of 41% females, women are overrepresented in the questionnaire. Compared to the real age distribution of physicians where 63% is between 30 and 60 years old and 35% is older than 60, the participant population seems to be younger as 67% is between 30 and 60 years old and 17% is older than 60. Therefore, the results reported in the current study are not generalizable to the broader physician population. While experts were consulted to create a scientifically robust questionnaire, the questions included in the questionnaire had to be generic since multiple specialties were targeted. The researchers are very aware that when asking about rare diseases, responses can vary according to specialty, previous knowledge, and in general the rare or even ultra-rare disease that the physicians were thinking about while filling out the questionnaire. Therefore, readers should be cautious when interpreting these results. In addition, the predetermined questionnaire response number (*n* = 400) was not reached as only 295 physicians completed the questionnaire, hindering statistical analysis and interpretation. Finally, it is important to report that the study was performed at a moment in time where the Belgian and European rare disease landscape was changing fast and perceptions might change over time. Therefore, we believe it would be interesting to re-examine physicians’ knowledge, awareness and information needs on rare diseases within a couple of years.

## Conclusion

Even though a lot of physicians are confronted with one or more rare disease patients during their career, the average level of awareness regarding rare diseases is suboptimal. Most pediatricians and specialists have a good basic knowledge about rare diseases, but a lack of knowledge about rare diseases was identified in GPs. Physicians should be informed on where to find correct and trustworthy rare disease information in a specialty specific manner meeting the preferences of these physicians. Information on prevention and screening, rare diseases symptoms, differential diagnosis and patient referral might be most valuable for all physicians. While GPs only need patient-related rare disease information, specialists wish to receive additional information about available treatments and orphan drugs. Information channels most suitable for the distribution of rare disease information were found to be expert centers and continuous training sessions. However, the digital platform Orphanet has the potential to become the golden standard of rare disease information for all physicians if upgraded to a platform that allows patient symptoms and lab-test results as input data, providing different outputs including rare disease differential diagnosis with suggested additional diagnostic tests or questions to ask the patient, possible treatment options and contact details of experts, reference centers and patients’ associations. Such a tool might also be valuable for use during the academic medical training of physicians. In training courses that combine common disease and rare disease topics and use casuistry, students and attendees should be made aware of “red flags” that indicate the casus might be on a rare disease. “Red flag” attentiveness can redirect a diagnosis and facilitate correct diagnosis in the end. Using “red flags” in the academic education, continuous education and also in diagnostic tool development must be stimulated.

Concluding, to effectively support Belgian physicians in diagnosing rare diseases at an early stage, the academic medical education on rare diseases should be revised. Teaching methods should be focused more on casuistry and “red flags”, based on computer analysis of real world data. In addition, an upgraded Orphanet digital platform, meeting information needs of physicians, including but not limited to rare disease symptoms, reference centers and diagnostic tests, might be ideal to support correct and timely rare disease diagnosis. Optimally, the combination of revised academic medical education and an upgraded digital platform will results in less late and misdiagnosis of rare disease patients, when available leading to timely appropriate treatment and a better quality of life for these patients.

## Additional files

Additional file 1: Exploratory interviews. (DOCX 25 kb)

Additional file 2: Questionnaires. (DOCX 1648 kb)

## Abbreviations

EU: European Union
EURORDIS: European organization for rare diseases
GP: General practitioner
HCP: Health care professional
PED: Pediatrician
PHCP: Primary health care professional
QUAGOL: Qualitative Analysis Guide of Leuven
SAS: Statistical Analysis System
SP adult: Adult specialist – medical specialist treating adult patients
SP child: Pediatric specialist – medical specialist treating pediatric patients
UK: United Kingdom
USA: United States of America
WIV-ISP: Wetenschappelijk Instituut Volksgezondheid – Institut de Santé Publique
