# Supplementary material for: Information needs of physicians regarding the diagnosis of rare diseases: a questionnaire-based study in Belgium
Source: Orphanet J Rare Dis. 2019 May 4;14:99. doi: 10.1186/s13023-019-1075-8 (PMC6500578; doi:10.1186/s13023-019-1075-8)
Supplement: Supplementary file 1 — Exploratory interviews. (DOCX 25 kb) [file 13023_2019_1075_MOESM1_ESM.docx]

**Additional file 1: Exploratory interviews**

**I.I Dutch interview guide**

Interview GIDS

experts Zeldzame ziekten

1. ***Hoe kan, volgens u, “rare disease awareness” bijdragen aan het versnellen van de diagnose en het verminderen van het aantal misdiagnosen bij patiënten met zeldzame ziekten?***
   1. *In welke mate is er al “rare disease awareness” bij eerstelijnsartsen –dit zijn huisartsen en kinderartsen-, volgens u?*
   2. *Hoe verschilt de “rare disease awareness” bij specialisten van de awareness bij eerstelijnsartsen, volgens u?*
   3. *Is er, volgens u, een verschil tussen de “rare disease awareness” bij Vlaamse, Brusselse en Waalse artsen? Waarom wel/niet?*
2. ***Hoe kunnen, volgens u, de opleiding geneeskunde en navormingsactiviteiten bijdragen aan een hogere “rare disease awareness”?***
   1. *In welke mate vindt u dat de opleiding geneeskunde voor een voldoende grote en bruikbare basiskennis zorgt over zeldzame ziekten en de diagnose ervan? En over ultra-zeldzame ziekten?*
   2. *In welke mate vindt u dat navormingsactiviteiten bijdragen aan een voldoende grote en bruikbare basiskennis over zeldzame ziekten en de diagnose ervan? En over ultra-zeldzame ziekten?*
   3. *Is er op vlak van opleiding over zeldzame ziekten een verschil tussen Vlaanderen, Brussel en Wallonië vindt u? Waarom wel/niet?*
3. ***Hoe kunnen, volgens u, informatiebronnen specifiek over zeldzame ziekten bijdragen aan een hogere “rare disease awareness”?***
   1. *Welke informatiebronnen specifiek over zeldzame ziekten kent u? (Orphanet, Eurordis, RaDiOrg, Rare Disorders Belgium) Wat is de aard van deze informatiebronnen? (website, brochure, congres)*
   2. *Welke gebruikt u op de dag van vandaag om uw kennis over zeldzame ziekten te verhogen? Of om een betere diagnose te kunnen stellen?*
   3. *Welke zijn de voor- en nadelen van de huidig beschikbare informatiebronnen rond rare disease awareness?*
4. ***Hoe zou, volgens u, de perfecte informatiebron eruitzien die het bewustzijn of “rare disease awareness” bij artsen naar een hoger niveau kan tillen?***
5. *Hoe zou zo een perfecte informatiebron bij kunnen dragen tot een betere/snellere diagnose van zeldzame ziekten?*

**I.II French interview guide**

guide d’Interview

expert en maladies rares

***1. Comment est-ce que, selon vous, la « rare disease awareness » peut contribuer à accélérer le diagnostic et à diminuer le nombre de diagnostics fautifs des patients?***

1. *Dans quelle mesure la « rare disease awareness » existe déjà chez les médecins généralistes et pédiatriques ?*
2. *Comment est-ce que la “rare disease awareness” des spécialistes diffère de celle de médecins généralistes et pédiatriques ?*
3. *Selon vous, existe-t-il une différence en matière de “rare disease awareness” entre les médecins en Flandre, à Bruxelles et en Wallonie? Pourquoi ?*

***2. Comment est-ce que, selon vous, la formation de médecin et la formation permanente peuvent contribuer à une « rare disease awareness » plus élevée ?***

1. *Dans quelle mesure la formation actuelle de médecin assure une connaissance de base suffisante et utile concernant les maladies rares et le diagnostic de ces maladies? Et concernant les maladies ultra-rares ?*
2. *Dans quelle mesure est-ce que la formation permanente actuelle assure une connaissance de base suffisante et utile concernant les maladies rares et le diagnostic de ces maladies? Et concernant les maladies ultra-rares ?*
3. Est-ce que la formation de médecin en matière de maladies rares est différente entre la Flandre, Bruxelles et la Wallonie ?

***3. Comment est-ce que, selon vous, les sources d’information qui concernent les maladies rares, peuvent contribuer à une « rare disease awareness » plus élevée ?***

1. *Quelles sources d’information qui concernent les maladies rares connaissez -vous? (Orphanet, Eurordis, RaDiOrg, Rare Disorders Belgium) Quelle est la nature de ces sources d’information ? (site web, congres, brochure)*
2. *Quelles sources d’information utiliseriez-vous pour améliorer la connaissance des maladies rares ? Ou pour effectuer un diagnostic ?*
3. *Quelles sont les avantages et désavantages des sources d’information actuelles en matière de maladies rares ?*

***4. Quelle est, selon vous, la source d’information idéale qui peut augmenter le niveau de la « rare disease awareness » chez les médecins?***

1. *Comment est-ce que cette source d’information idéale peut contribuer à effectuer de façon plus rapide et correcte un diagnostic?*
