# Supplementary material for: Information needs of physicians regarding the diagnosis of rare diseases: a questionnaire-based study in Belgium
Source: Orphanet J Rare Dis. 2019 May 4;14:99. doi: 10.1186/s13023-019-1075-8 (PMC6500578; doi:10.1186/s13023-019-1075-8)
Supplement: Supplementary file 2 — Questionnaires. (DOCX 1648 kb) [file 13023_2019_1075_MOESM2_ESM.docx]

**Additional file 2: Questionnaires**

**II.I Dutch questionnaire**


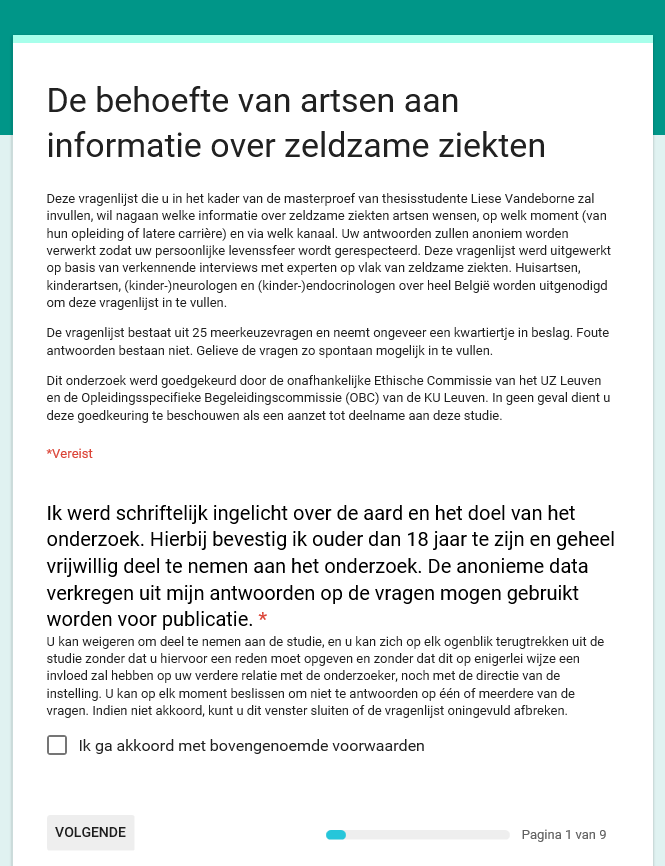


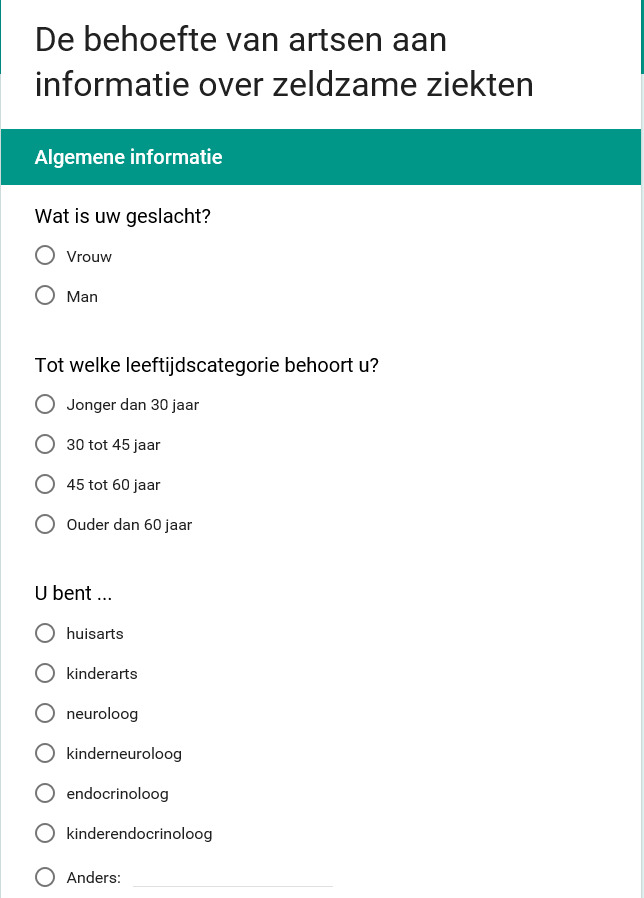


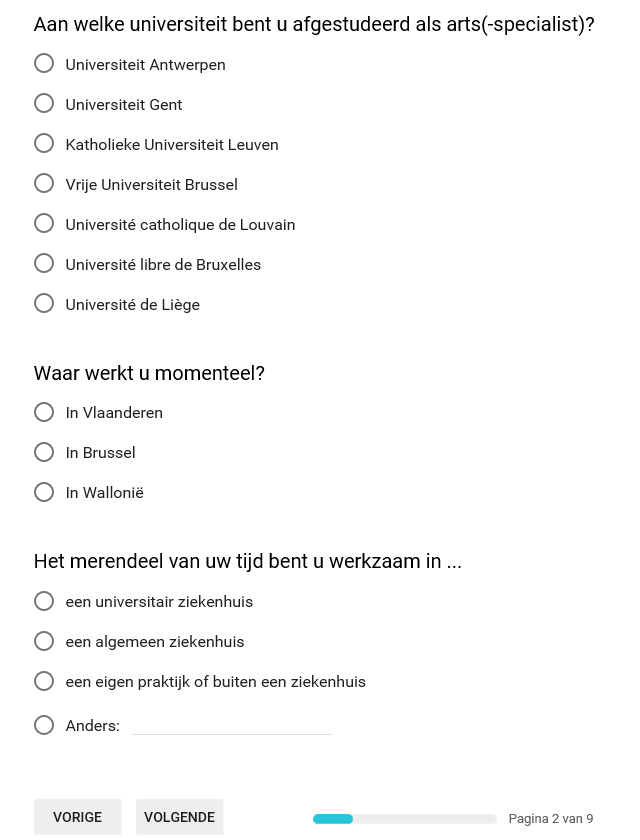


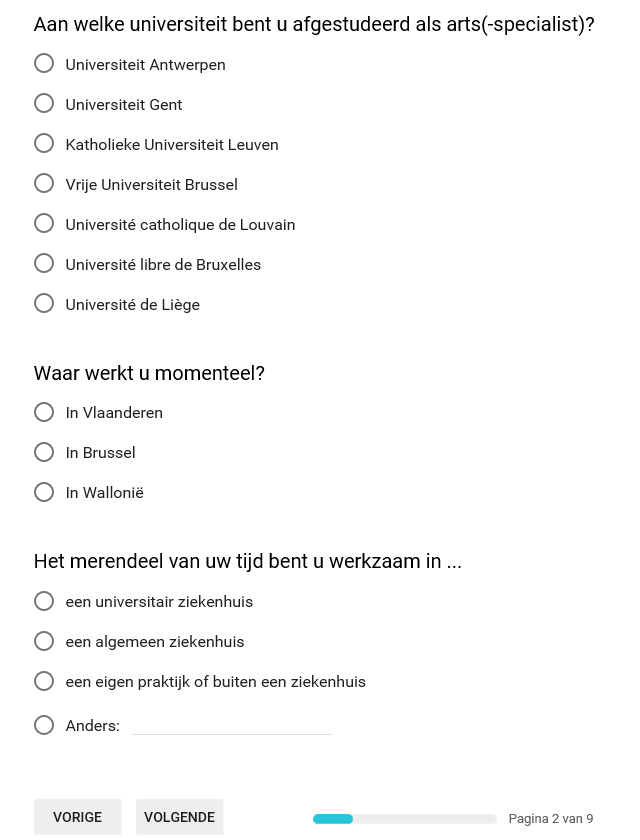


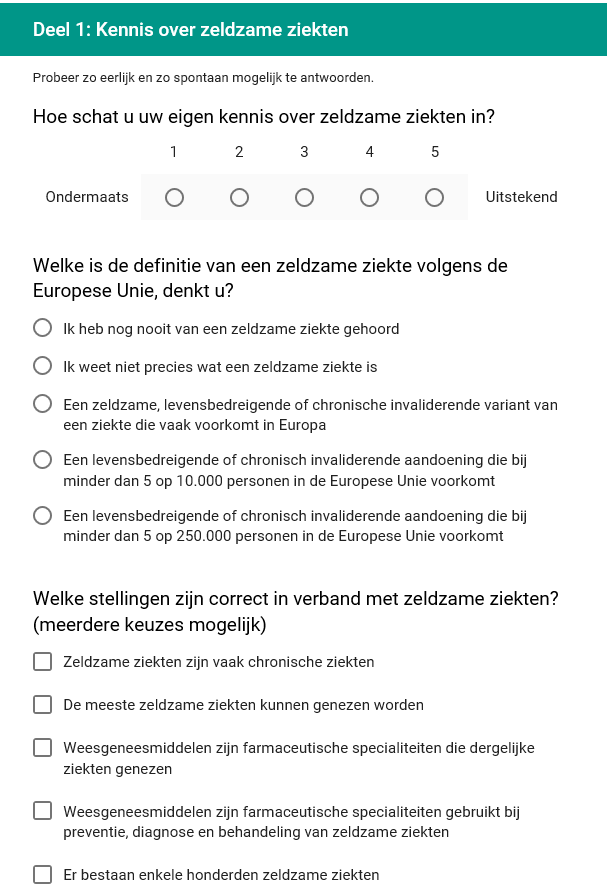


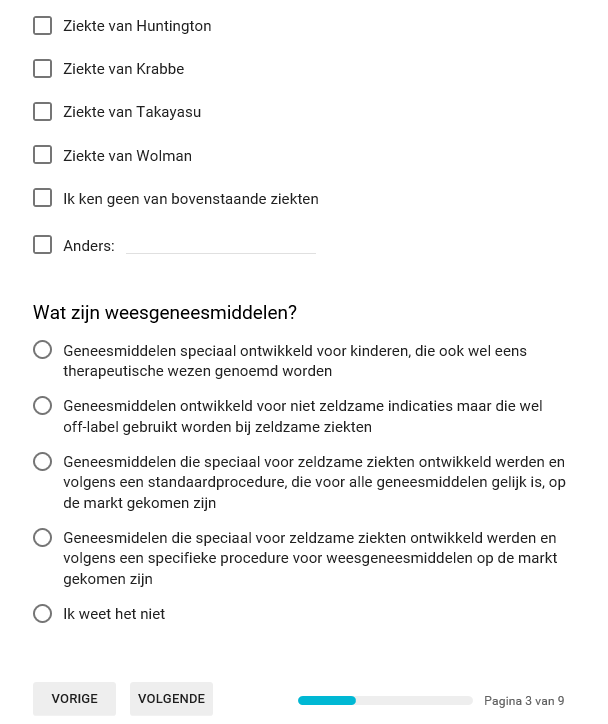

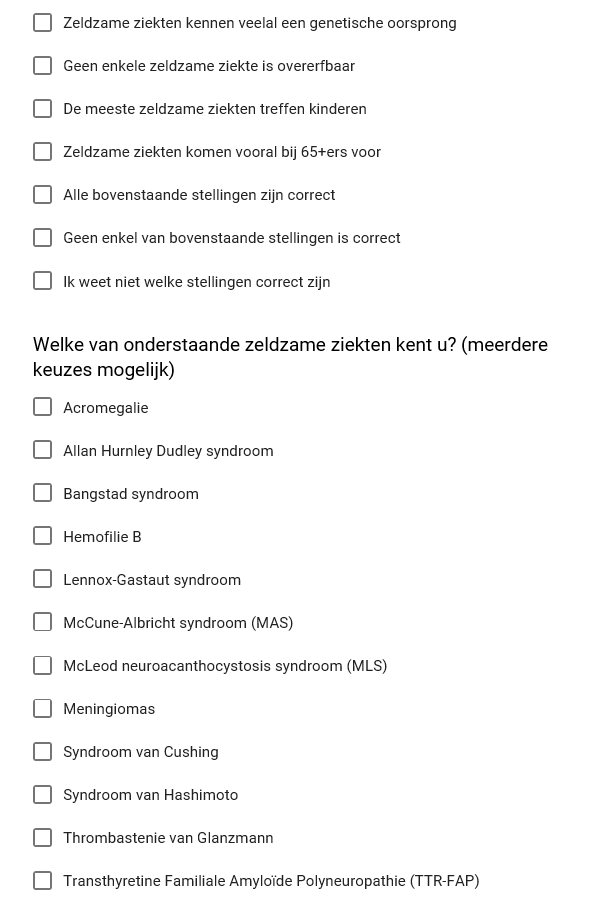


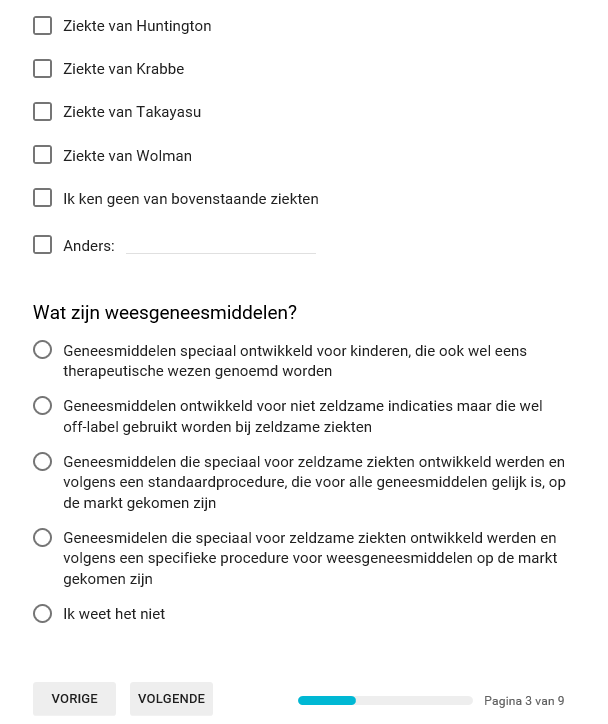


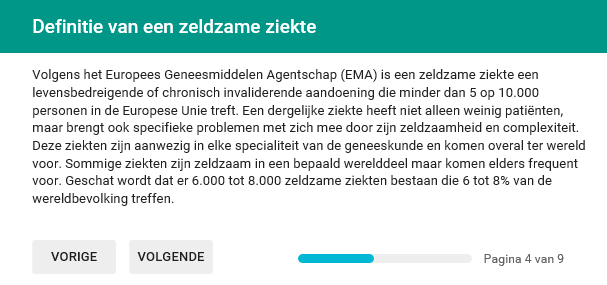


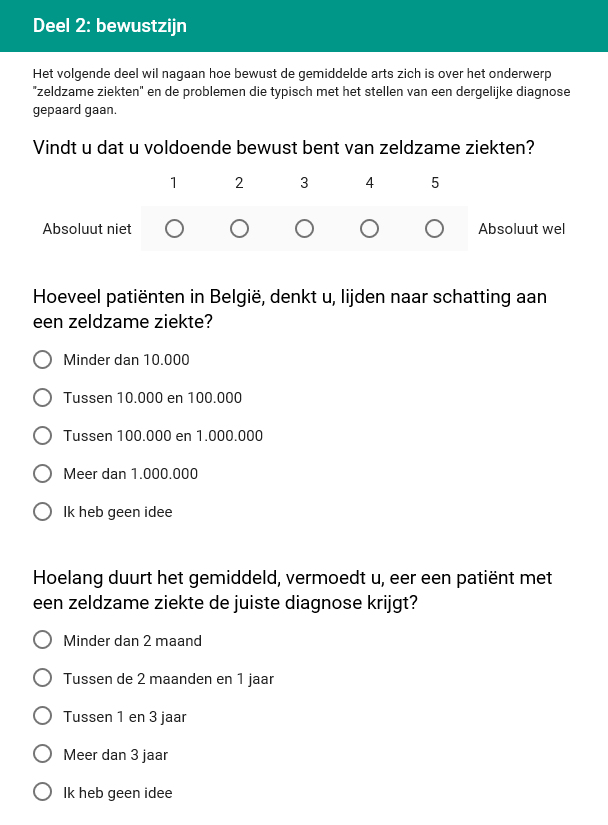


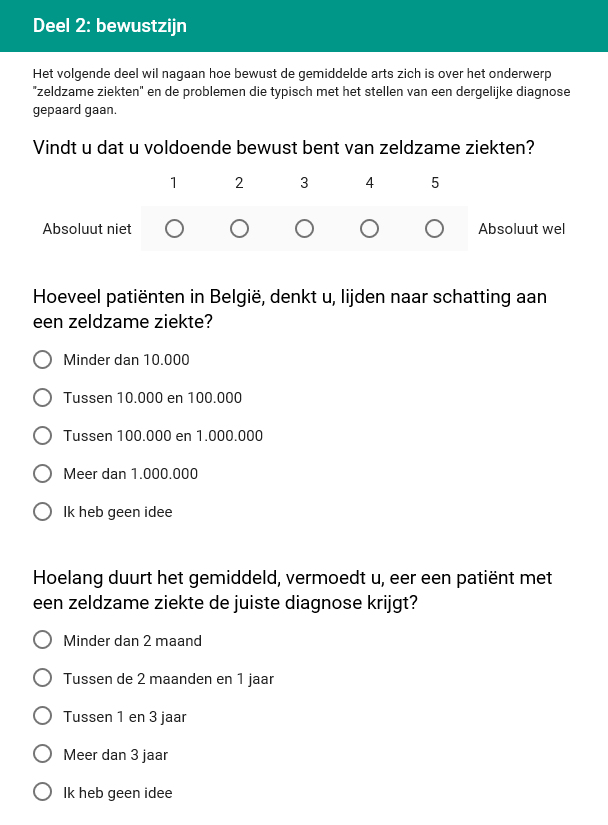


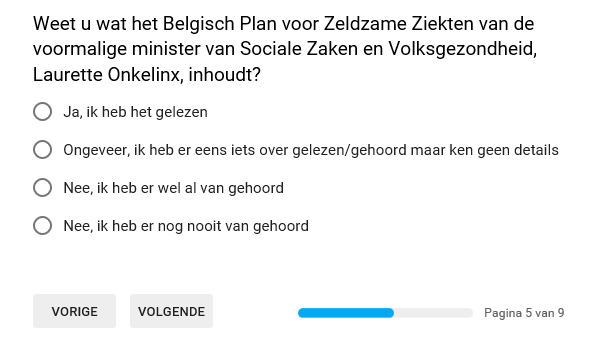


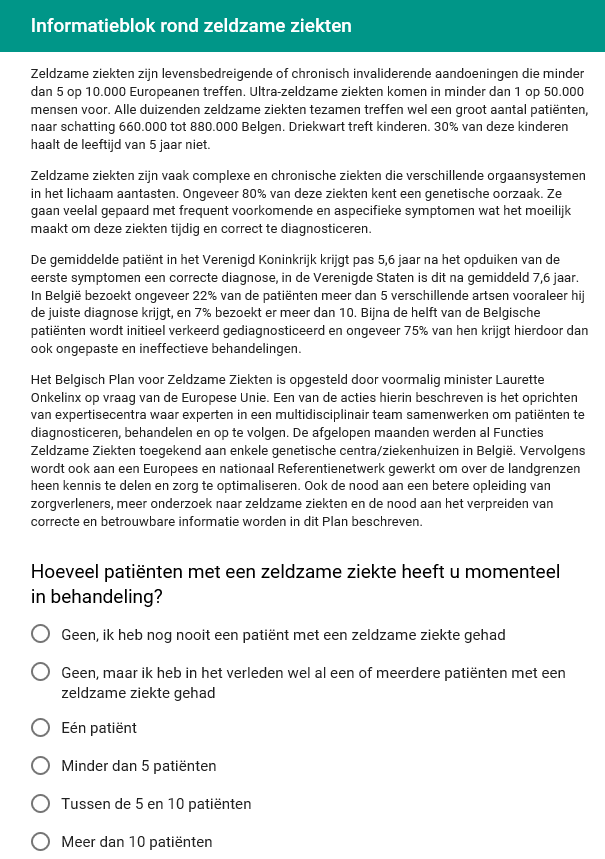


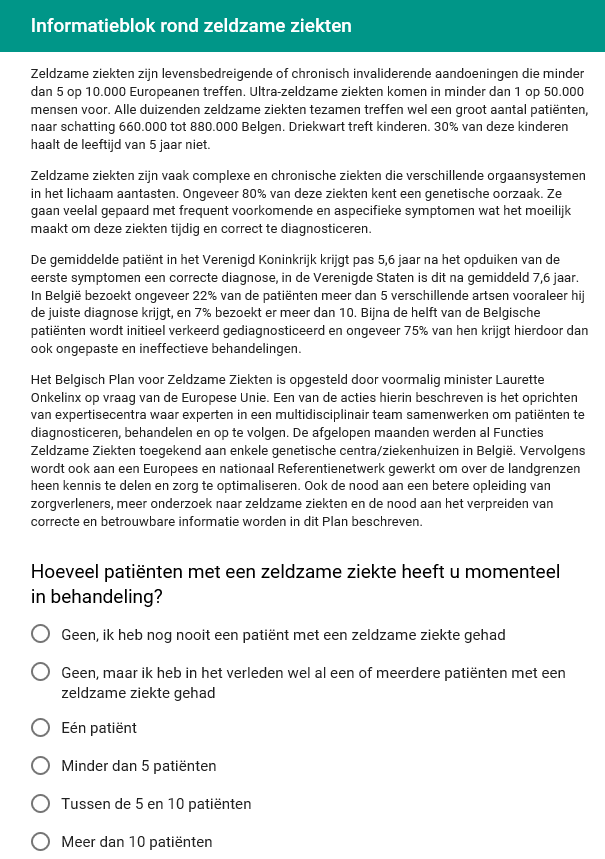


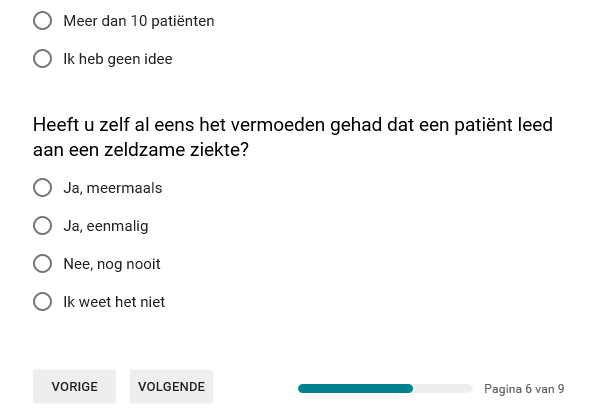


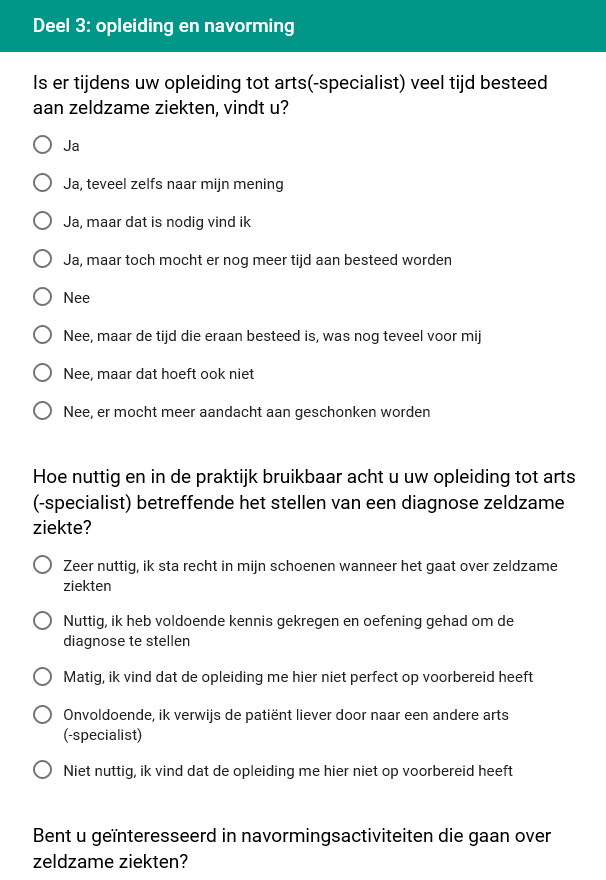


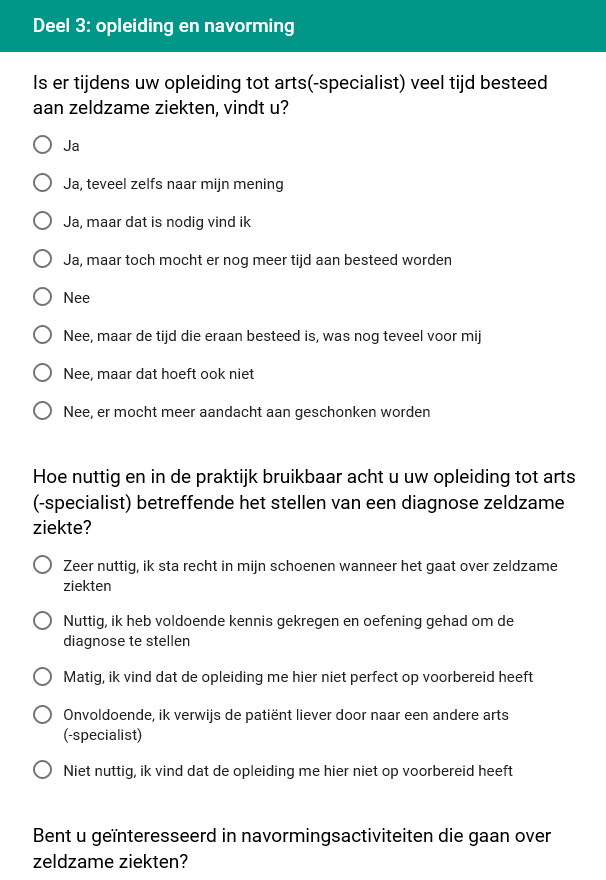


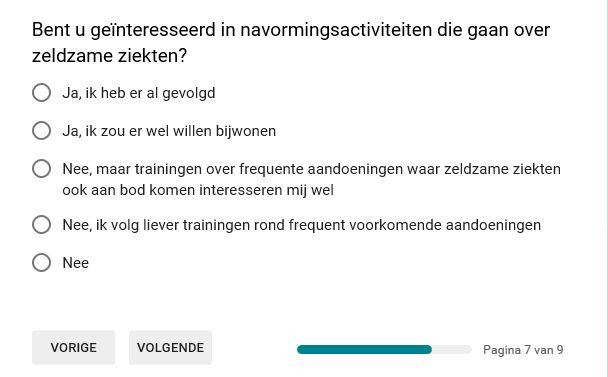


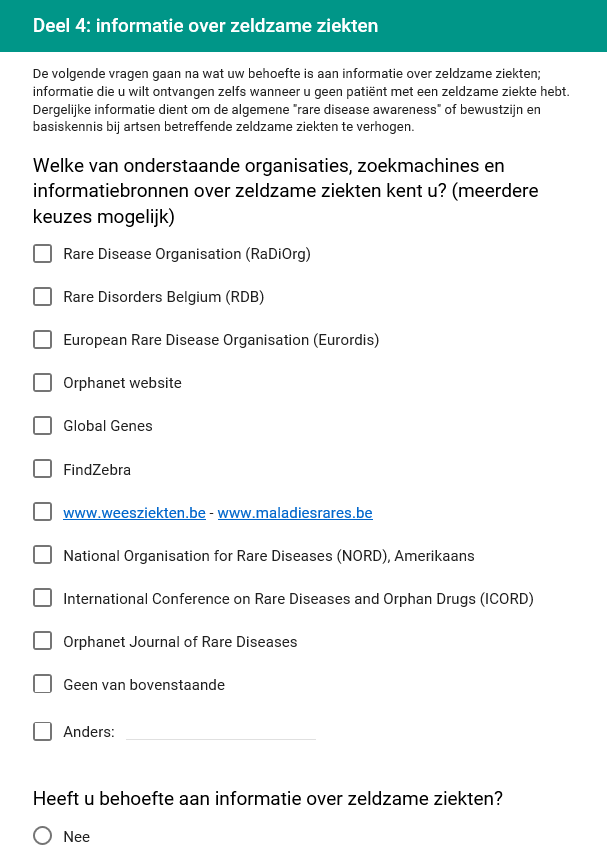


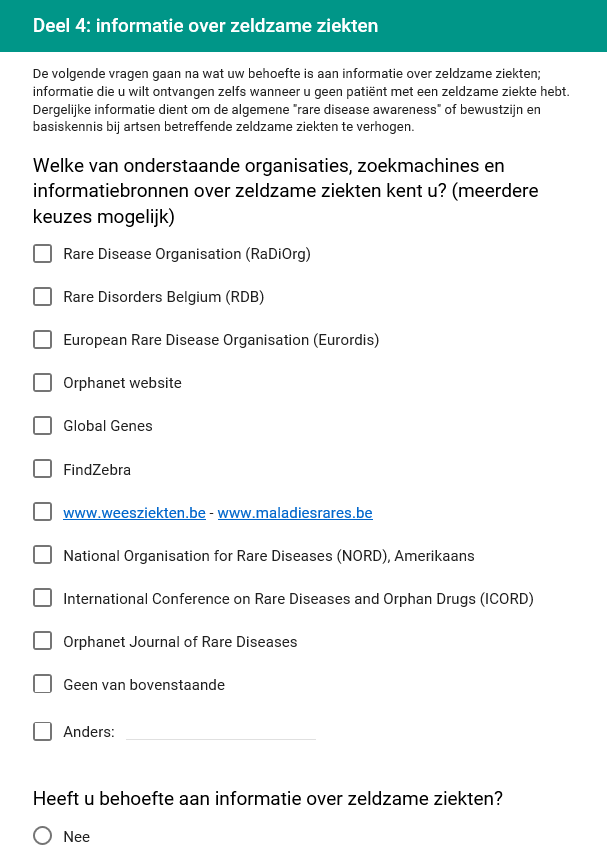


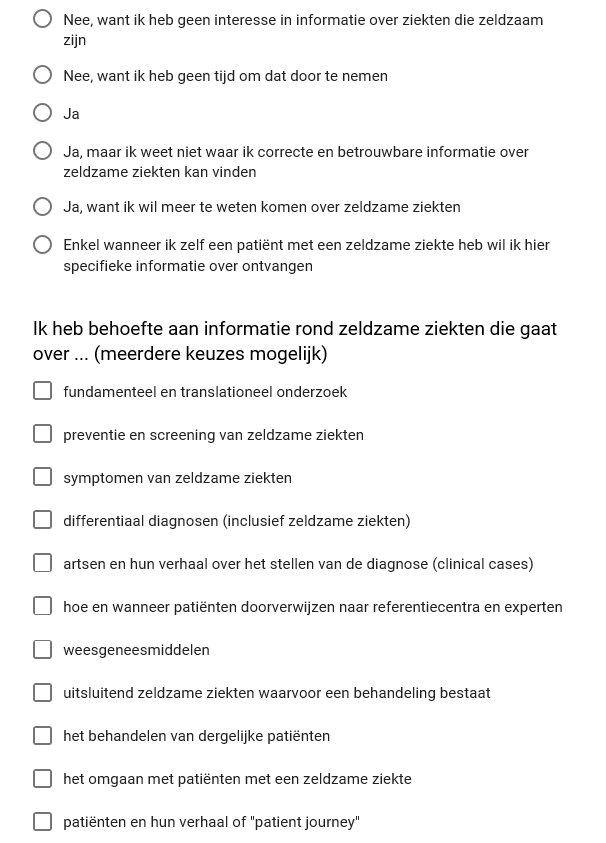


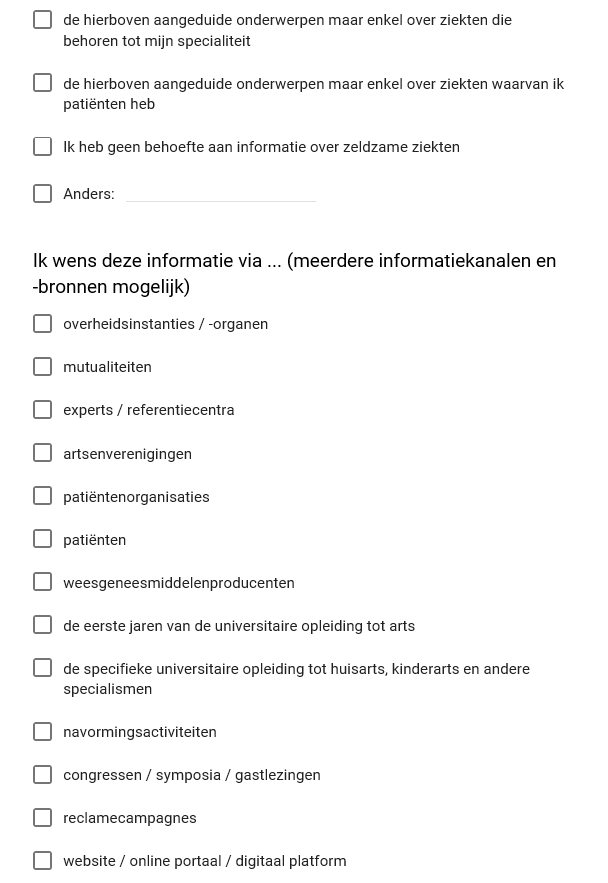


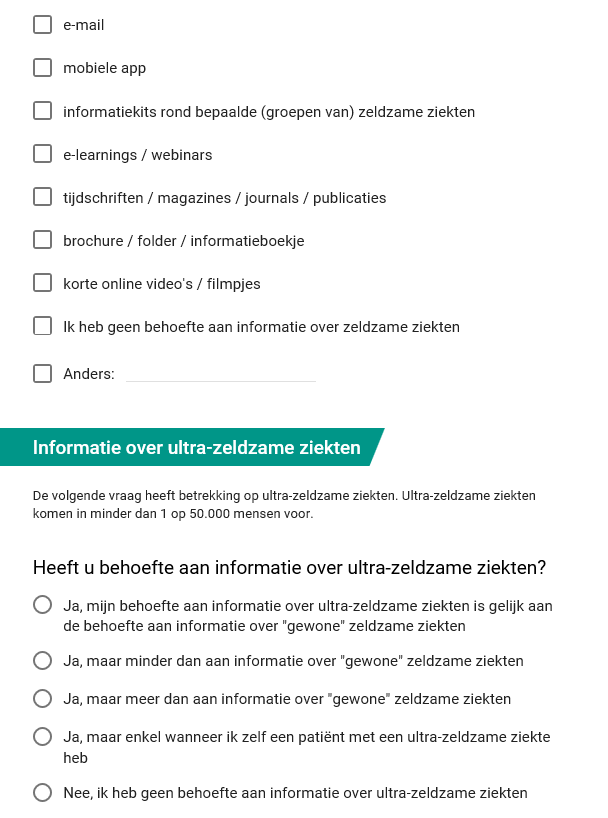


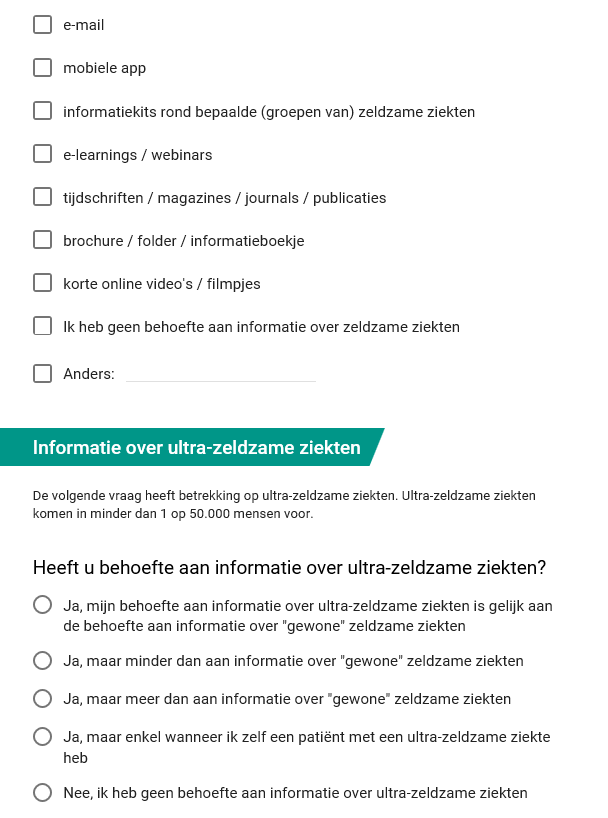


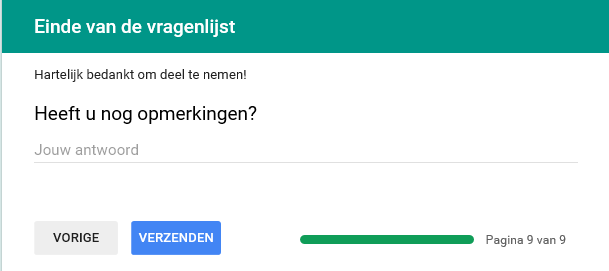


**II.II French questionnaire**


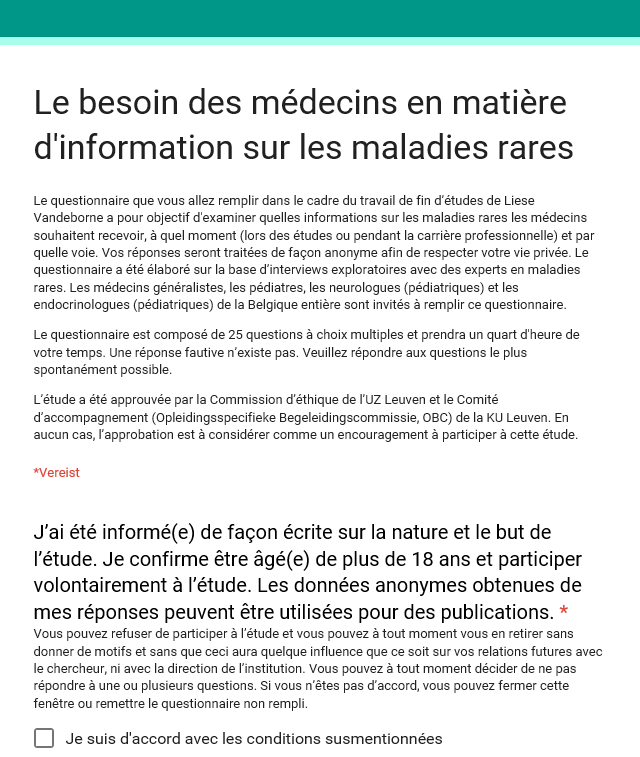


*nécessaire


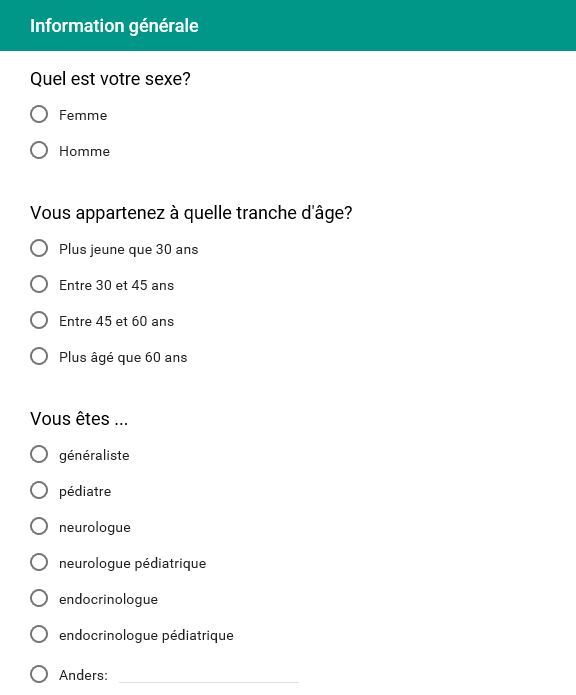


**Autre**:


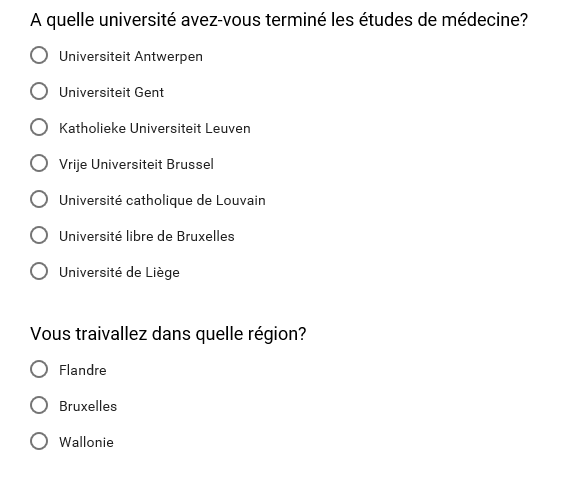


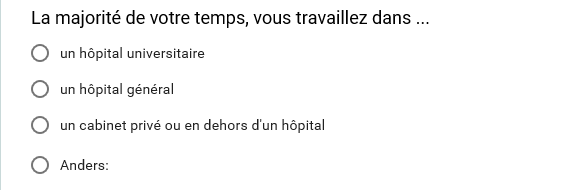


**Autre**:


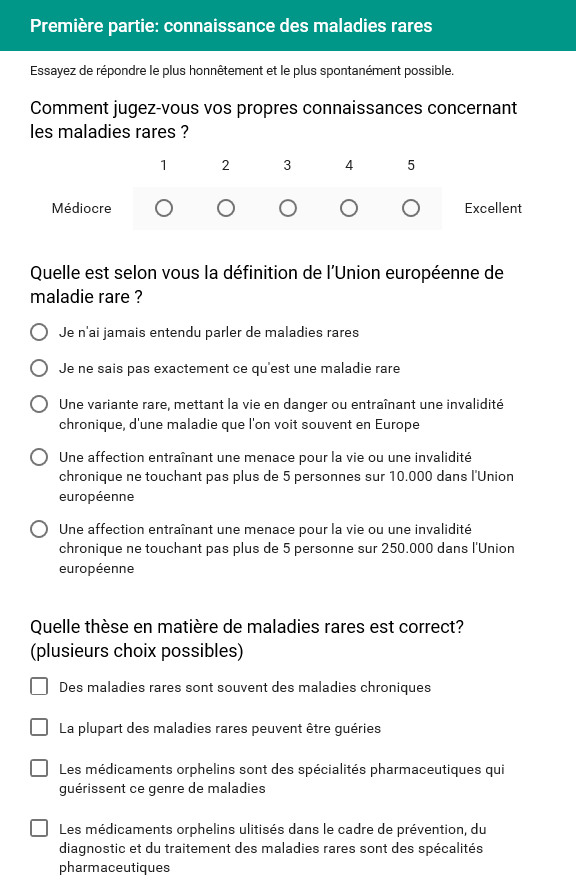


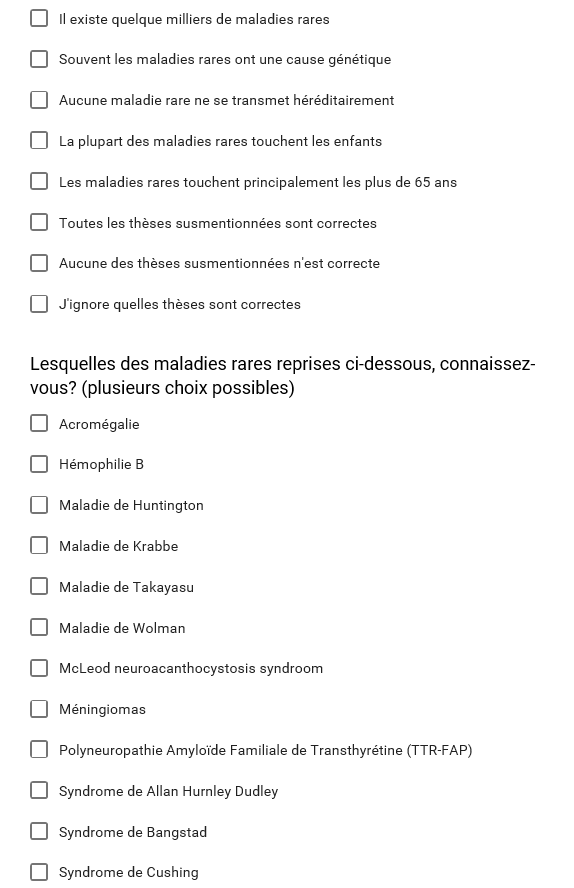


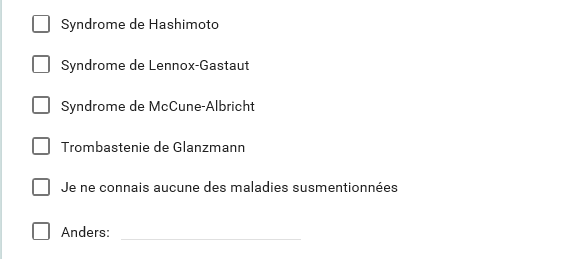


**Autre**:


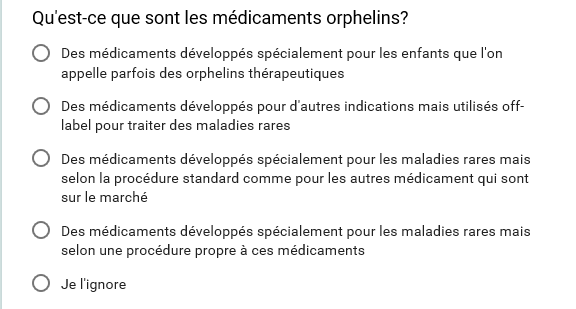


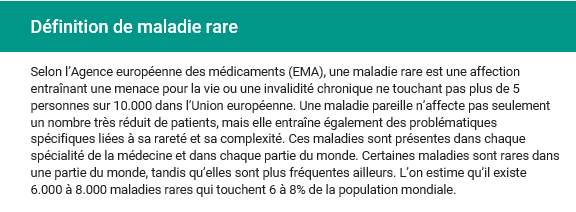


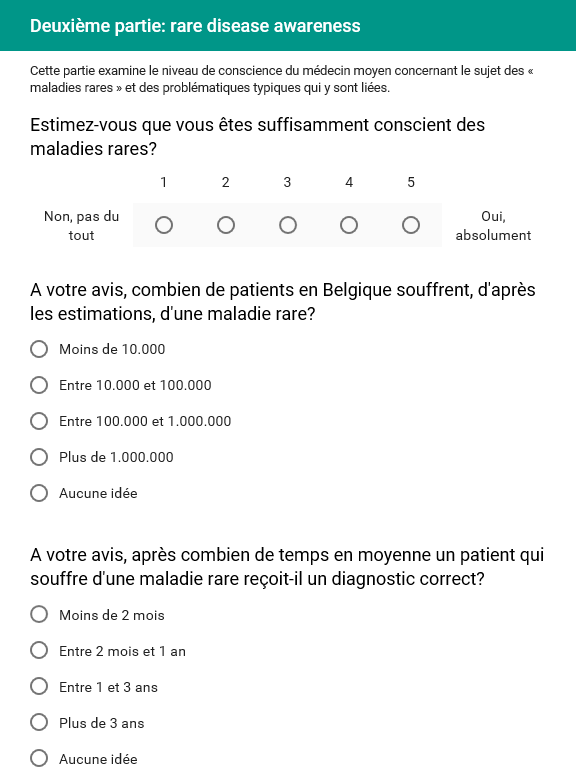


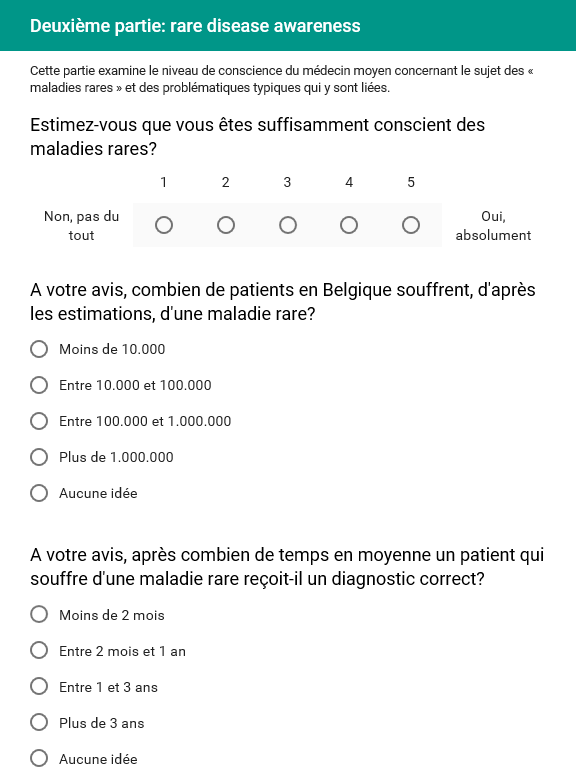


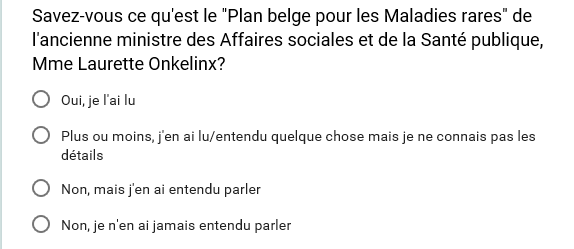


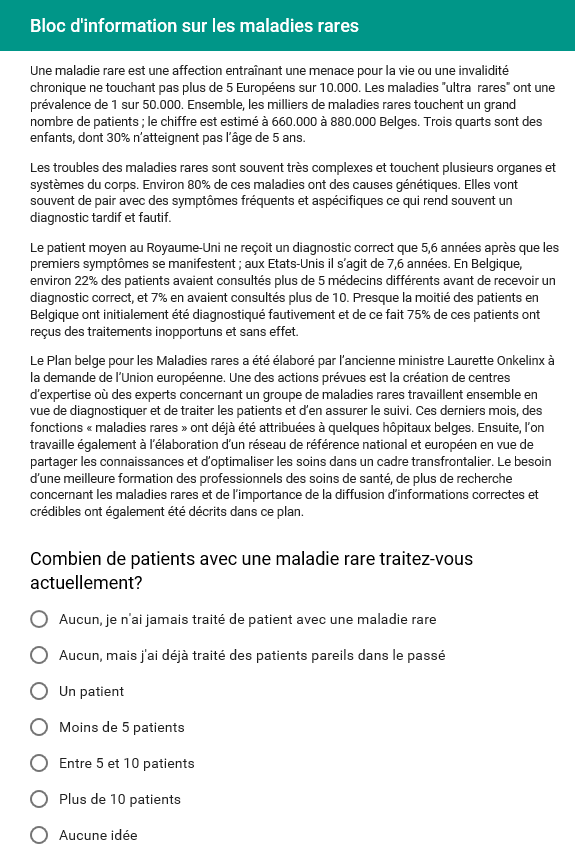


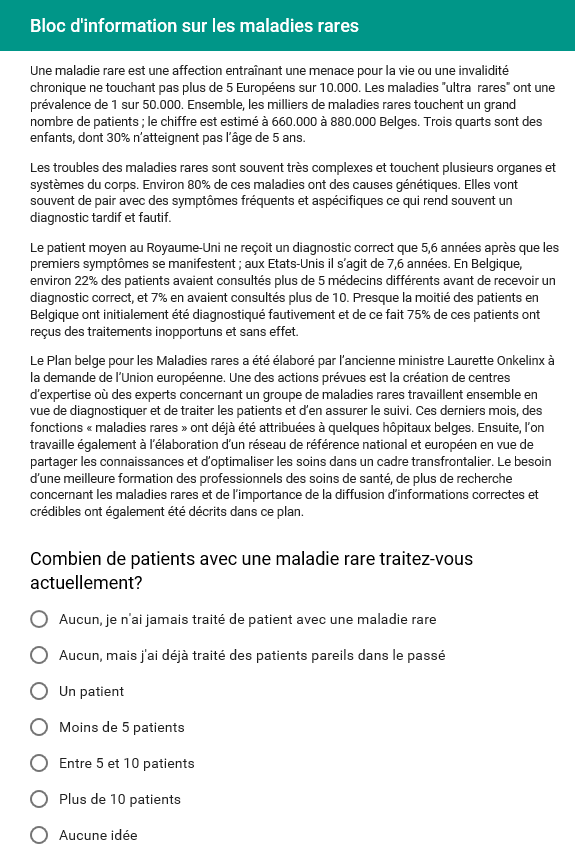


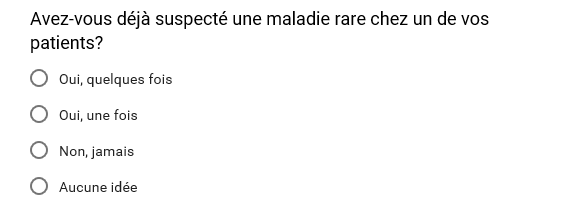


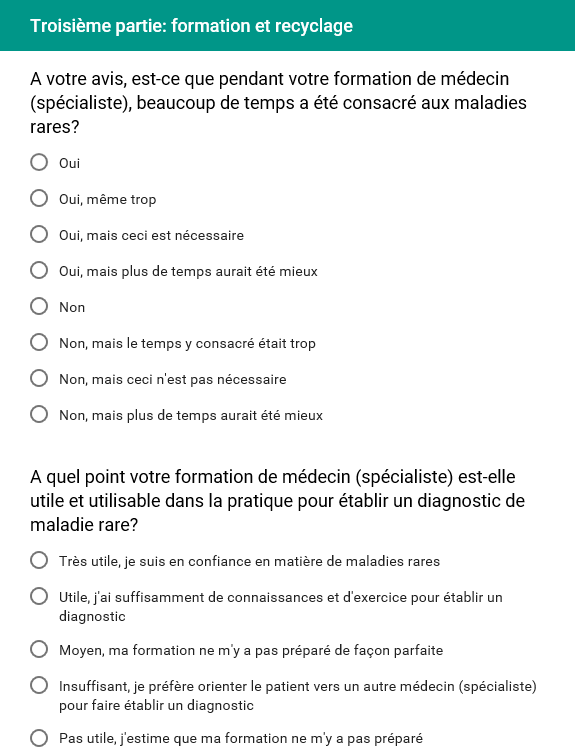


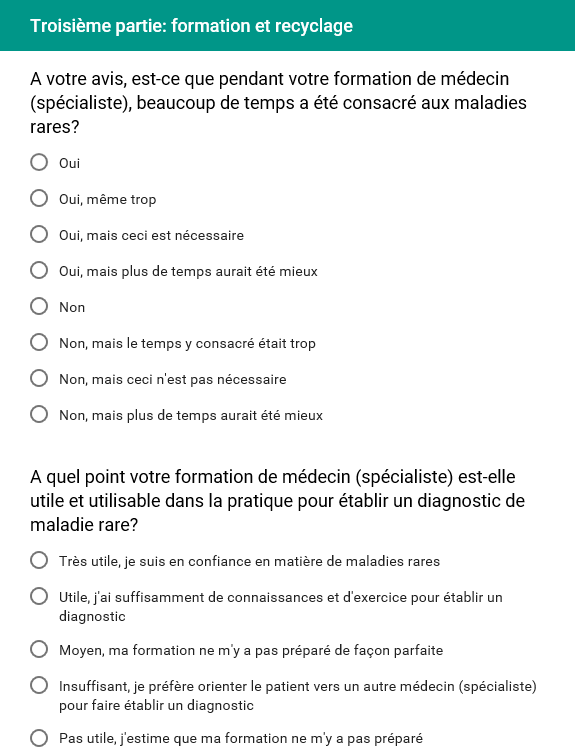


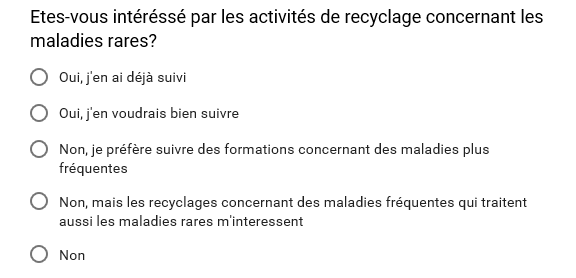


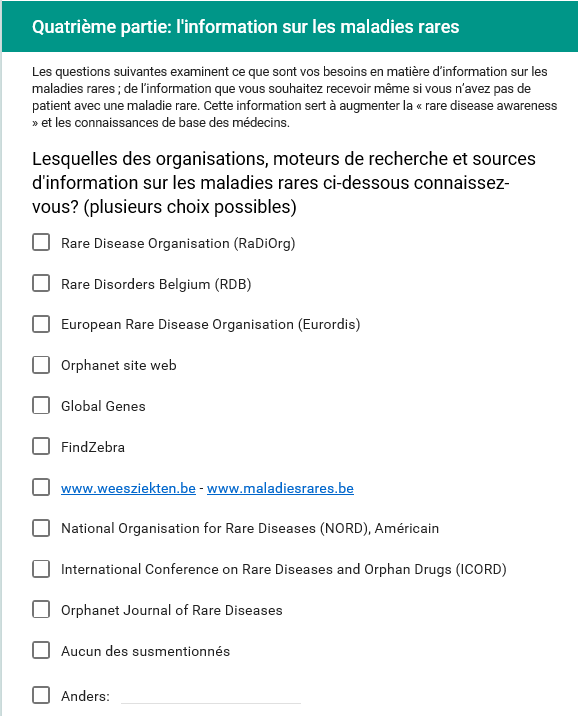


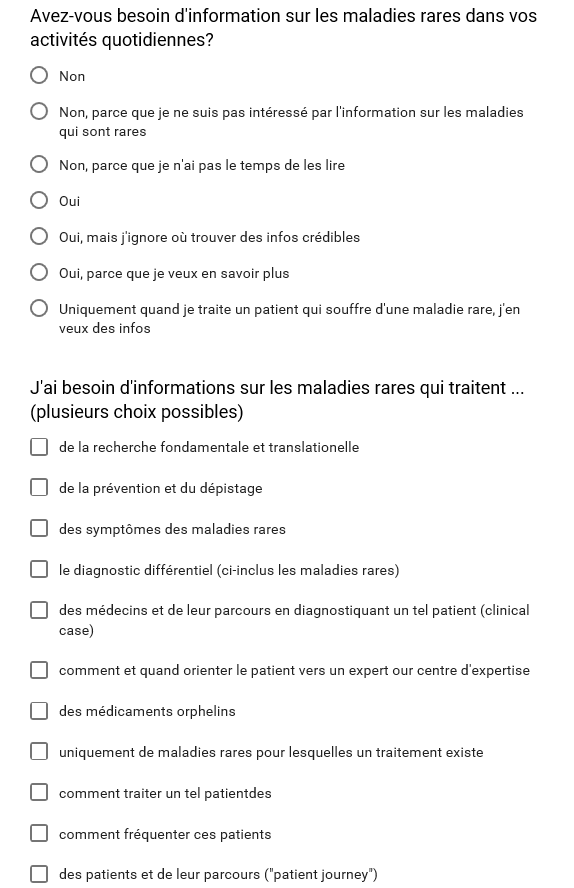


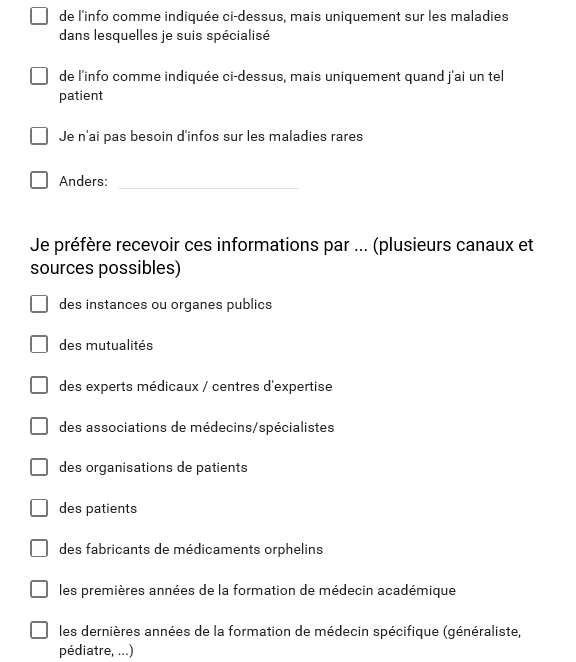


**Autre**:


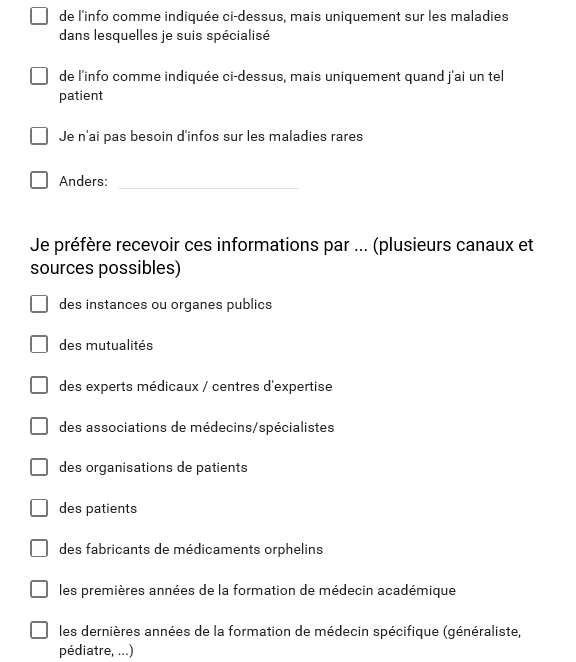


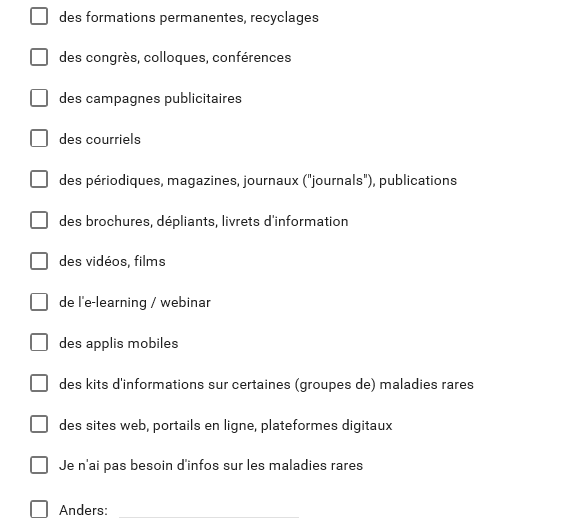


**Autre**:


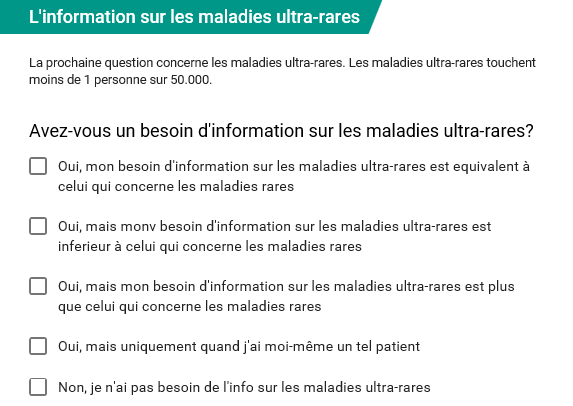


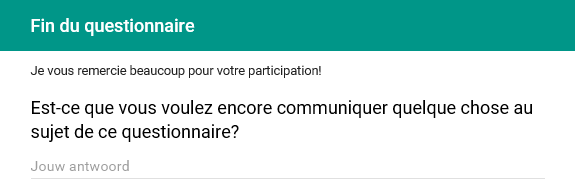


Votre réponse:
